# Evaluation and clinical significance of cyclin-dependent kinase5 expression in cervical lesions: a clinical research study in Guangxi, China

**DOI:** 10.1186/s40001-016-0222-0

**Published:** 2016-07-12

**Authors:** Deng-Hua Pan, Mei-Lin Zhu, Xiao-Miao Lin, Xing-Gu Lin, Rong-Quan He, Yan-Xin Ling, Shi-Tao Su, Madushi Mihiranganee Wickramaarachchi, Yi-Wu Dang, Kang-Lai Wei, Gang Chen

**Affiliations:** Department of Pathology, First Affiliated Hospital of Guangxi Medical University, 6 Shuangyong Road, Nanning, 530021 Guangxi Zhuang Autonomous Region China; Department of Children Rehabilitation Medicine, Guangxi Maternal and Child Health Hospital, 225 Xinyang Road, Nanning, 530003 Guangxi Zhuang Autonomous Region China; Center for Genomic and Personalized Medicine, Guangxi Medical University, 22 Shuangyong Road, Nanning, 530021 Guangxi Zhuang Autonomous Region China

**Keywords:** Cyclin-dependent kinase5, Cervical cancer, Chronic cervicitis, Condyloma acuminate, IHC

## Abstract

**Background:**

Studies have been reported that cyclin-dependent kinase5 (CDK5) was associated with the development of several cancers. However, the relationship between CDK5 level and clinicopathological factors is still poorly understood in cervical diseases. The aim of the current study was to investigate the expression of CDK5 and its clinical significance in variant cervical lesions.

**Methods:**

Immunohistochemistry (IHC) was used to detect CDK5 expression in 54 cases of chronic cervicitis, 42 cases of condyloma acuminate (CA), 38 cases of carcinoma in situ, and 360 cases of cervical cancers [adenocarcinoma, *n* = 63; squamous cell carcinoma (SCC), *n* = 263; adenosquamous carcinoma, *n* = 34]. The clinicopathological characteristics in relation to CDK5 were examined by Pearson’s Chi-square test.

**Results:**

The positive rates of CDK5 were 27.8, 31.0, 50, 54.0, 58.8, and 62.7 % in chronic cervicitis, CA, carcinoma in situ, adenocarcinoma, adenosquamous carcinoma and SCC, respectively. Statistically analysis showed that CDK5 expression in cervical cancer tissues was higher than non-cervical cancer tissues (inflammation and CA) (*P* < 0.001). The overexpression of CDK5 was significantly correlated with lymph node metastasis (*r* = 0.317; *P* < 0.001), histological type (*r* = 0.198; *P* < 0.001), FIGO stage (*r* = 0.358; *P* < 0.001), TNM stage (*r* = 0.329; *P* < 0.001) and pathological grade (*r* = 0.259; *P* < 0.001) in cervical lesions evaluated by Pearson’s Chi-square test. Furthermore, the positive relationships were found between CDK5 and lymph node metastasis (*P* < 0.001), FIGO stage (*P* < 0.001), TNM stage (*P* < 0.001) and pathological grade (*P* < 0.001) in SCC. CDK5 was positively interrelated to TNM stage (*P* = 0.017) in adenosquamous carcinoma.

**Conclusions:**

CDK5 may play a vital role in the development of cervical cancer, which may be a marker for the diagnosis, therapy and prognosis of cervical cancer.

## Background

According to GLOBOCAN 2012, cervical cancer is the fourth most frequent cancer after breast, colorectal and lung cancer in women and there were an estimated 527,600 new cervical cancer cases and 265,700 deaths worldwide in 2012 [1]. In China, it was demonstrated that there were 98.9 per 100, 000 female diagnosed as cervical cancer and 30.5 per 100, 000 died from this disease in 2015 [2]. Also, a project exploring the cancer survival in 67 countries reported that the 5-year survival range was less than 50 % to more than 70 % of the world and the rate of 5-year survival was increased (from 40 to 60 %) in China [3].

Currently, surgical removal and radiotherapy are the main treatments for cervical cancer. Unfortunately, a tiny percentage of patients with cervical cancer in early stage disease can receive timely diagnosis and efficient treatment. Worse still, patients have a poor survival rate, with an expected 5-year survival of less than 10 % [4]. A Thai case study indicated that high burden and other factors may influence the screening services of cervical cancer [5]. These outcomes strongly suggest the requirement of innovative research to explore new approaches to the diagnosis and treatment in the early stage of cervical cancer. Cyclin-dependent kinase 5 (CDK5), a proline-directed serine/threonine kinase, belongs to the CDK family activated by its regulators p35 or p39 [6]. Studies have reported that CDK plays a chief role in the cell cycle by regulating cell growth, differentiation, movement and apoptosis, and is closely related to the occurrence, development and metastasis of tumor [7–10].

However, the relationship between CDK5 level and clinicopathological factors is still poorly understood in cervical diseases. To our knowledge, only two studies have investigated the relationship between CDK5 and HeLa cells, a type of cervical cancer cell line due to infection by human papilloma virus (HPV), which found that CDK5 might have an effect on the apoptosis of HeLa cells [11, 12]. To date, no study has been available to investigate the expression and clinicopathological significance of CDK5 expression in cervical cancer tissues. Thus, in this study, we aimed to investigate the expression of CDK5 and its clinical significance in variant cervical lesions.

## Methods

### Materials

We retrospectively analyzed 512 operated cases at the first affiliated hospital of Guangxi Medical University over a period of January, 2011–December, 2014. Fifty-six cases were excluded for the following criteria: If the size of the tissue was too small (diameter <0.5 cm), we excluded it to keep the clinical evidence for diagnosis. Moreover, tissues falling out of the slide were also excluded in the process of immunohistochemistry. And only 360 cases of cervical cancer and 134 cases of non- cervical cancer were selected in the paper.

Cases of cervical cancer were staged according to the International Federation of Gynecology and Obstetrics (FIGO) 2009 [13] (stage I, *n* = 248; stage II, *n* = 16; stage III–IV, *n* = 96). Pathology grading was according to the World Health Organization (WHO) criteria [14] (grade I, *n* = 28, grade II, *n* = 157, grade III, *n* = 141) and tumor node metastasis (TNM) stage was according to American Joint Committee on Cancer (AJCC) Cervix Uteri Cancer Staging 7th edition [15] (stage 0, *n* = 38, stage I, *n* = 248, stage II–IV, *n* = 112). Ninety six of 360 cervical cancer cases had lymph node metastases. None of the cases received radiotherapy or chemotherapy. All the patients agreed to participate in this study and institutional review board-approved written cones were provided by patients before study entry. The study was approved by the Ethics Committee of the First Affiliated Hospital of Guangxi Medical University (Nanning, Guangxi, China) (NO. 2013/2016-KY-GUOJI-136).

### Immunohistochemical staining to detect CDK5

In this study, immunohistochemistry (IHC) was performed on tissue-microarrays. The pathological diagnosis was confirmed after the tissue was removed by surgical operation or tissue biopsy, and the tissue was fixed with neutral-buffered formalin (10 %) and the fixation time was 24–48 h according to the tissue size. IHC staining for CDK5 was performed on 4 µm thick paraffin sections obtained from all cervical tissues in paraffin embedded blocks and conducted HE staining and histological observation. Slices were dealt with high pressure hotfix, immunohistochemical PV and DAB chromogenic and hematoxylin retying, hydrochloric acid-alcohol differentiation, anhydrous ethanol dehydration, neutral gum sealing piece. Immunostaining for CDK5 was performed using CDK5 rabbit monoclonal antibody (Abeam, Clone ID: EP715Y; 1:50 dilution) and immunohistochemical kit was purchased from Beijing jinqiao biological co. LTD. All steps for immunohistochemical detection of CDK5 were strictly operated according to the kit instructions. PBS of 0.01 mol/L (pH7.4) was used as a negative control. The sections were examined under a light microscope and claybank granules showed in the tissue were considered as positive CDK5 expression.

### Immunohistochemical scoring

CDK5 expression was divided into negative group and positive group. The immunohistochemical staining was assessed by three authors (Yi-Wu Dang, Kang-Lai Wei and Gang Chen) and an agreement regarding controversial case was reached at a multithreaded microscope. CDK5 score was determined by both intense staining and positive cell amounts in tumor tissues. CDK5 expression was classified semi quantitatively according to the following criteria: no staining was recorded as 0; weak staining presenting as focal or fine granular was noted as 1; strong staining with linear or cluster pattern was 2 and diffuse, intense staining was regarded as 3. The positive cell amounts in the tissue sample ranged from 0 to 3 in percentage: no staining was 0; <30 % was 1; 30–70 % was 2 and >70 % was 3. The samples were categorized as positive and negative based on the sum of the scores as follows: 0–2: negative (−); 3–6: positive (+).

### Statistical analysis

The statistical package SPSS version 20.0 (SPSS Inc, Chicago, illions, USA) was used to study the relationship between the differential expression of CDK5 and clinicopathological parameters of cervical tissues. Two independent-samples Chi-square test and Kruskal–Wallis *H* test were used to analyze the differences between groups. The correlation between CDK5 and clinicopathological characteristics should be assessed by Pearson’s Chi-square test. A *P* value of <0.05 was considered statistically significant.

## Results

### The expression of CDK5 in cervical tissues

A total of 494 cervical specimens which were selected, including 54 cases of chronic cervicitis, 42 cases of CA, 38 cases of carcinoma in situ and 360 cases of cervical cancers (adenocarcinoma, *n* = 63; squamous cell carcinoma (SCC), *n* = 263; adenosquamous carcinoma, *n* = 34). The patients aged from 19 to 82 years (median 44 years). Clinicopathological information of the patients was available in Tables 1, 2, 3, 4 and 5.

**Table 1:** Relationship of CDK5 expression with other clinicopathological variables

Characteristic	Total number	No. patients, stratified by CDK5		*Z*	*P* value
		Negative (228)	Positive (266)		
Age				−1.557	0.120^a^
≤44	263	130 (49.4 %)	133 (50.6 %)		
>44	231	98 (42.4 %)	133 (57.6 %)		
Lymph node metastasis				−5.998	<0.001^a^
Negative	264	128 (48.5 %)	136 (51.5 %)		
Positive	96	13 (13.5 %)	83 (86.5 %)		
Histological type				32.490	<0.001^b^
Chronic cervicitis	54	39 (72.2 %)	15 (27.8 %)		
CA	42	29 (69.0 %)	13 (31.0 %)		
Carcinoma in situ	38	19 (50.0 %)	19 (50.0 %)		
Adenocarcinoma	63	29 (46.0 %)	34 (54.0 %)		
SCC	263	98 (37.3 %)	165 (62.7 %)		
Adenosquamous carcinoma	34	14 (41.2 %)	20 (58.8 %)		
FIGO stage				−5.998	<0.001^a^
I–II	264	128 (48.5 %)	136 (51.5 %)		
III–IV	96	13 (13.5 %)	83 (86.5 %)		
TNM stage				52.911	<0.001^b^
0	38	19 (50.0 %)	19 (50.0 %)		
I	248	128 (51.6 %)	120 (48.4 %)		
II–IV	112	13 (11.6 %)	99 (88.4 %)		
Pathological grade				23.604	<0.001^b^
I	28	16 (57.1 %)	12 (42.9 %)		
II	157	77 (49.0 %)	80 (51.0 %)		
III	141	34 (24.1 %)	107 (75.9 %)		

*CA* condyloma acuminates, *SCC* squamous cell carcinoma, *FIGO* International Federation of Gynecology and Obstetrics, *TNM* tumor node metastasis

^a^Two independent-samples Chi-square test was performed

^b^Kruskal–Wallis H test was performed

**Table 2:** The correlation between CDK5 expression and clinicopathological factors in cervical tissues

Variable	*r*	*P*
Lymph node metastasis	0.317	<0.001
Histological type	0.198	<0.001
FIGO stage	0.358	<0.001
TNM stage	0.329	<0.001
Pathological grade	0.259	<0.001

*FIGO* International Federation of Gynecology and Obstetrics, *TNM* tumor node metastasis

**Table 3:** Relationship of CDK5 expression with other clinicopathological variables in adenocarcinoma

Characteristic	Total number	No. patients, stratified by CDK5		*Z*	*P* value
		Negative (29)	Positive (34)		
Age				−2.316	0.021^a^
≤44	27	17 (63.0 %)	10 (37.0 %)		
>44	36	12 (33.3 %)	24 (66.7 %)		
Lymph node metastasis				−1.269	0.205^a^
Negative	18	6 (33.3 %)	12 (66.7 %)		
Positive	45	23 (51.1 %)	22 (48.9 %)		
FIGO stage				−1.269	0.205^a^
I–II	45	23 (51.1 %)	22 (48.9 %)		
III–IV	18	6 (33.3 %)	12 (66.7 %)		
TNM stage					
I	43	23 (53.5 %)	20 (46.5 %)	2.983	0.084^a^
II–IV	20	6 (30.0 %)	14 (70.0 %)		
Pathological stage				5.455	0.065^b^
I	10	6 (60.0 %)	4 (40.0 %)		
II	38	20 (52.6 %)	18 (47.4 %)		
III	15	3 (20.0 %)	12 (80.0 %)		

*FIGO* International Federation of Gynecology and Obstetrics, *TNM* tumor node metastasis

^a^Two independent-samples Chi square test was performed

^b^Kruskal–Wallis H test was performed

**Table 4:** Relationship of CDK5 expression with other clinicopathological variables in SCC

Characteristic	Total number	No. patients, stratified by CDK5		*Z*	*P* value
		Negative (98)	Positive (165)		
Age				−1.167	0.243^a^
≤44	138	56 (40.6 %)	82 (59.4 %)		
>44	125	42 (33.6 %)	83 (66.4 %)		
Lymph node metastasis				−5.785	<0.001^a^
Negative	193	92 (47.7 %)	101 (52.3 %)		
Positive	70	6 (8.6 %)	64 (91.4 %)		
FIGO stage				−5.785	<0.001^a^
I–II	193	92 (47.7 %)	101 (52.3 %)		
III–IV	70	6 (8.6 %)	64 (91.4 %)		
TNM stage					
I	181	92 (50.8 %)	89 (49.2 %)	45.53	<0.001^a^
II–IV	82	6 (7.3 %)	76 (92.7 %)		
Pathologicl grade				16.909	<0.001^b^
I	18	10 (55.6 %)	8 (44.4 %)		
II	119	57 (47.9 %)	62 (52.1 %)		
III	126	31 (24.6 %)	95 (75.4 %)		

*FIGO* International Federation of Gynecology and Obstetrics, *TNM* tumor node metastasis

^a^Two independent-samples Chi-square test was performed

^b^Kruskal–Wallis H test was performed

**Table 5:** Relationship of CDK5 expression with other clinicopathological variables in adenosquamous carcinoma

Characteristic	Total number	No. patients, stratified by CDK5		*Z*	*P* value
		Negative (14)	Positive (20)		
Age				−0.122	0.918^a^
≤44	19	8 (42.1 %)	11 (57.9 %)		
>44	15	6 (40.0 %)	9 (60.0 %)		
Lymph node metastasis				−1.857	0.120^a^
Negative	26	13 (50.0 %)	13 (50.0 %)		
Positive	8	1 (12.5 %)	7 (87.5 %)		
FIGO stage					
I–II	26	13 (50.0 %)	13 (50.0 %)	−1.857	0.120^a^
III–IV	8	1 (12.5 %)	7 (87.5 %)		
TNM stage					
I	24	13 (54.2 %)	11 (45.8 %)	5.518	0.019^a^
II–IV	10	1 (10.0 %)	9 (90.0 %)		

*ND* no data, *FIGO* International Federation of Gynecology and Obstetrics, *TNM* tumor node metastasis

^a^Two independent-samples Chi-square test was performed

**Fig. 1:**
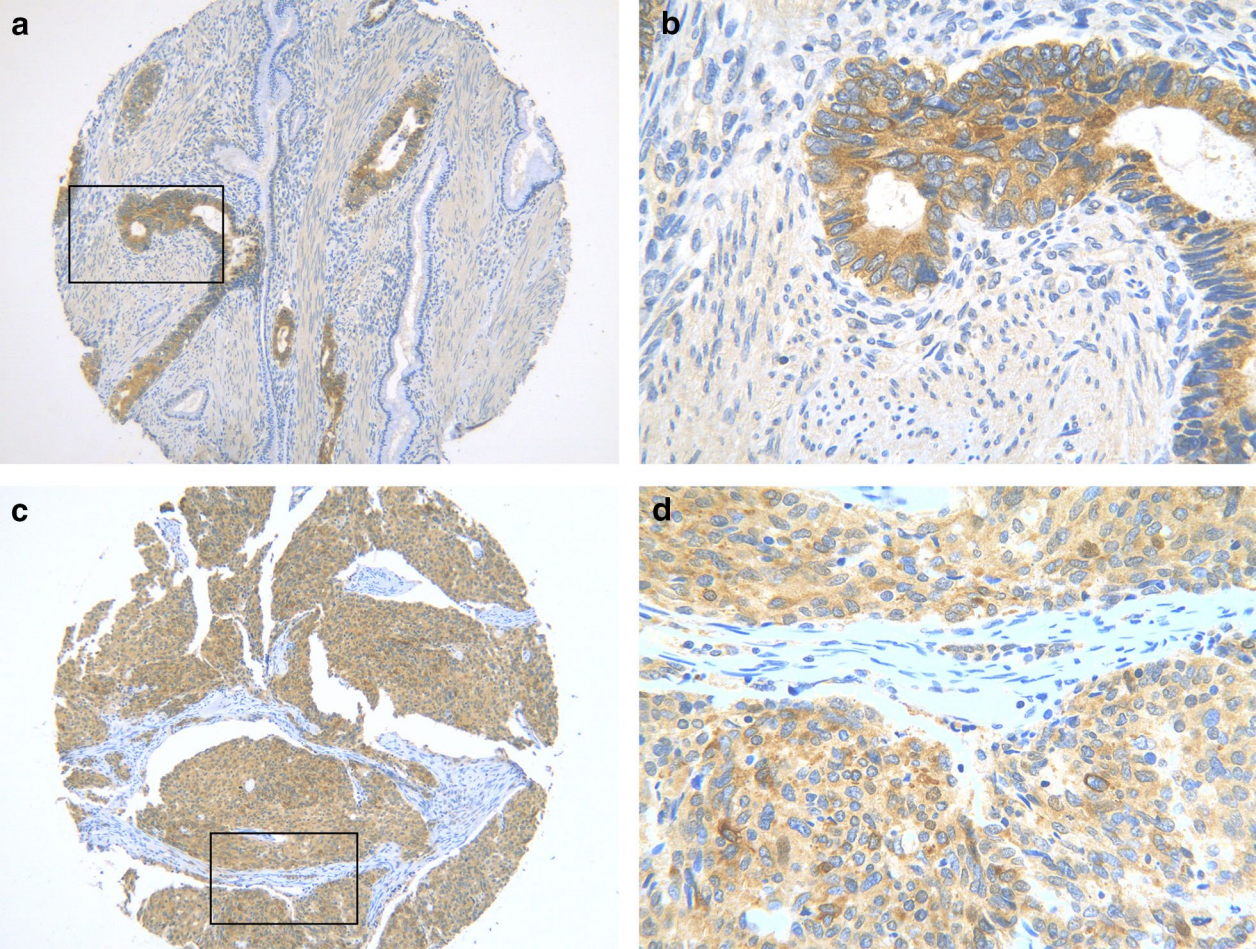
Immunohistochemical analysis of CDK5 expression in cervical cancer. **a** and **b** showed that CDK5 expressed strongly in adenocarcinoma. The area of the *square* in **a** represented **b**. **c** and **d** showed strong CDK5 expression in squamous carcinoma. The area of the *square* in **c** represented **d**.The original magnification of **a** and **c** was ×100. The original magnification of **b** and **d** was ×400

**Fig. 2:**
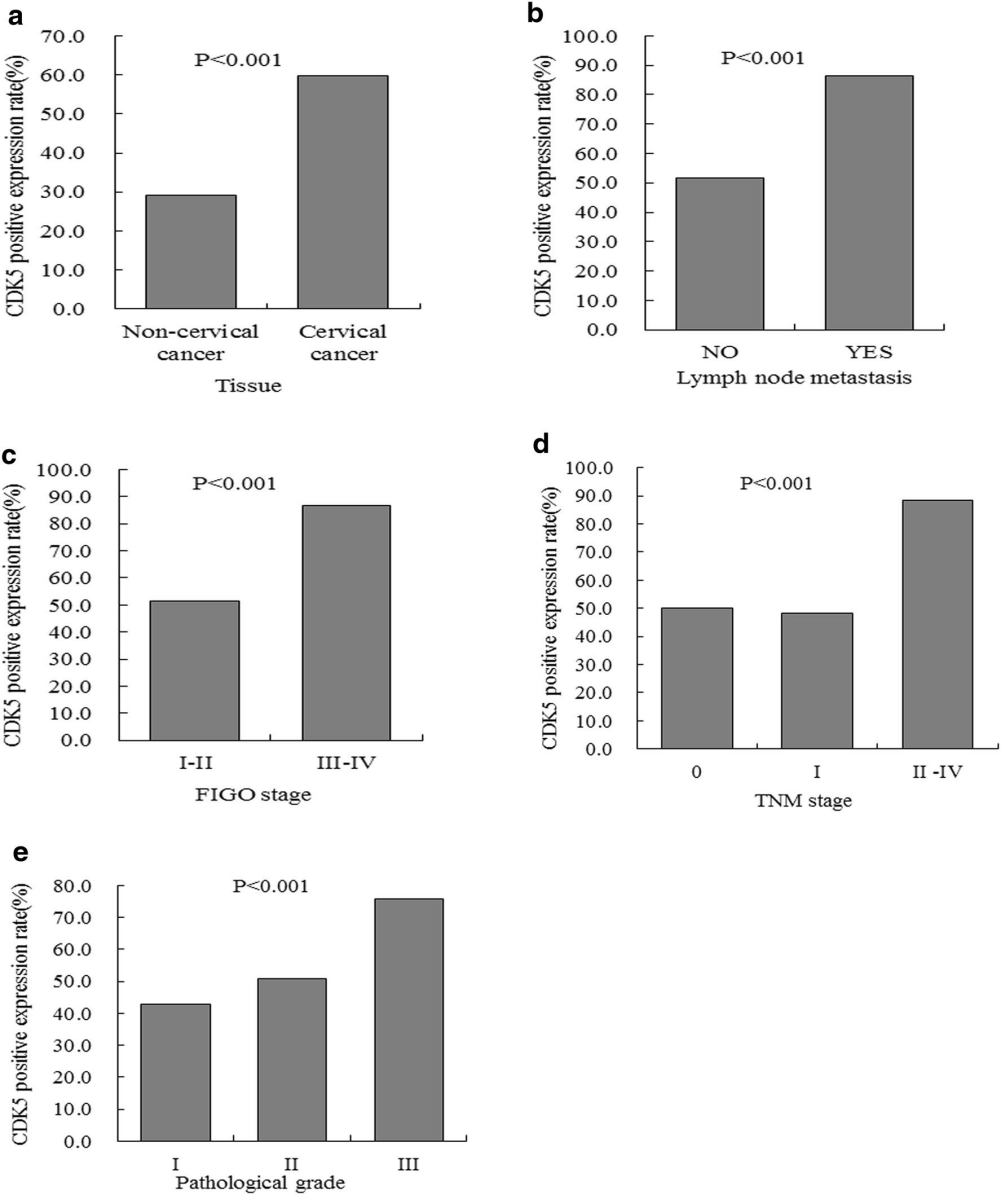
Relationship between CDK5 expression and some clinicopathological features in cervical tissues. **a** Tissue; **b** Lymph node metastasis; **c** FIGO stage; **d** TNM stage; **e** Pathological grade

The CDK5 expression was detected in 494 cases and 53.8 % tissues showed positive CDK5 expression. The pattern of CDK5 expression revealed by immunohistochemical analysis was illustrated in Fig. 1. The positive rates of CDK5 were 27.8, 31.0, 50, 54.0, 58.8, 62.7 % in chronic cervicitis, CA, carcinoma in situ, adenocarcinoma, adenosquamous carcinoma and SCC, respectively (Table 1). The expression of CDK5 was lower in non-cervical cancer tissues (29.2 %) compared with cervical cancer tissues (59.8 %) (*P* < 0.001) (Fig. 2a). CDK5 expression in the group with lymph node metastasis (86.5 %) was higher than that in the non-lymph node metastasis group (51.5 %) (*P* < 0.001) (Fig. 2b), indicating that CDK5 up- regulation may be relative to the carcinogenesis and progression of cervical cancer. The positive expression rate of CDK5 in advanced FIGO stage (86.5 %) was higher than early FIGO stage (51.5 %) (*P* < 0.001) (Fig. 2c). The expression of CDK5 in advanced TNM stage (stage II–IV) grading (88.4 %) was higher than stage 0 (50.0 %), stage I (48.4 %) (*P* < 0.001) (Fig. 2d). CDK5 positive expression level with pathological grade III (75.9 %) was higher than pathological grade I (42.9 %) and pathological grade II (51.0 %) (*P* < 0.001) (Fig. 2e).

### The correlation between CDK5 expression and clinicopathological factors in cervical tissues

The correlations between CDK5 and selected variables were shown in Table 2. CDK5 had a positive relationship with lymph node metastasis (*r* = 0.317; *P* **<** 0.001). Further, the relationships between CDK5 with histological type(*r* = 0.198; *P* **<** 0.001), FIGO stage(*r* = 0.358; *P* **<** 0.001), TNM stage(*r* = 0.329; *P* **<** 0.001) and pathological grade(*r* = 0.259; *P* **<** 0.001) had also been found in this analysis.

### Relationship between CDK5 expression and clinicopathological factors in different histological type of cervical cancer

Table 3 showed the relationships of CDK5 expression with clinicopathological variables in adenocarcinoma. CDK5 had a relationship with age (*P* = 0.021). Also, CDK5 expression had no association with lymph node metastasis, FIGO stage, TNM stage and pathological grade (all *P* **>** 0.05) in adenocarcinoma.

Table 4 showed the relationships of CDK5 expression with clinicopathological variables in SCC. CDK5 expression in patients without lymph node metastasis (52.3 %) was lower than with lymph node metastasis (91.4 %) (*P* **<** 0.001). In advanced TNM stage, CDK5 expression (92.7 %) was higher than early TNM stage (49.2 %) (*P* **<** 0.001). In early FIGO stage, the expression of CDK5 (52.3 %) was lower than advanced FIGO stage (91.4 %) (*P* **<** 0.001). The expression of CDK5 in different pathological grades was statistically significantly different (*P* **<** 0.001). The positive relationships were found between CDK5 and lymph node metastasis (*P* **<** 0.001), FIGO stage (*P* **<** 0.001), TNM stage (*P* **<** 0.001) and pathological grade (*P* **<** 0.001) in SCC.

Table 5 showed the relationships of CDK5 expression with clinicopathological variables in adenosquamous carcinoma. In advanced TNM stage, CDK5 expression (90.0 %) was higher than early TNM stage (45.8 %) in adenosquamous carcinoma (*P* = 0.019). TNM stage was positively interrelated to CDK5 (*P* = 0.019) in adenosquamous carcinoma.

## Discussion

In this study, we investigated the differential expression level of CDK5 in patients with cervical cancer and precancerous lesions so as to clarify the correlation between CDK5 and carcinogenesis and progression of cervical cancer.

Cervical cancer is one of the most common gynecological cancers, which has constituted a serious threat to the life and health of women [16, 17]. The complex pathogenesis of cervical cancer has not been clearly defined nowadays. And it is believed that the tumor genesis is a process of multifactor, polygenic and multi-step, which is still an attractive area worth exploring [18].

The abnormal regulation of cell cycle is known to play an essential role in the genesis of tumor. CDK is a class of serine/threonine protein kinases, which participate in the regulation of cell cycle. CDK5 is a member of CDKs family, but in comparison with the other CDK, it is not a cyclin-dependent kinase, nor directly is involved in the regulation of the cell cycle. Previous studies of CDK5 mainly focused on the neurological disorders, such as Alzheimer’s disease [19]. In recent years, studies have found that CDK5 was not only expressed in neurons, but also turned out to be of particular importance in non-neuronal cells, for instance: immune cells, endothelial cells, epithelial cells, tumor cells and so on. And in some studies, CDK5 was associated with the development of several cancers, such as lung cancer [20], breast cancer [21], prostate cancer [22] and neuroendocrine thyroid cancer [23]. These findings suggested that CDK5 could be of importance in the cancers tissues.

In this study, we primarily used IHC method to detect the expression of CDK5 in cervical cancer tissues and precancerous lesions, and analyzed its potential role in the tumor genesis of cervical cancer. Our results showed that CDK5 expression level was remarkably higher in cervical cancer tissues (59.8 %) compared with non-cancer tissues (29.2 %) (*P* **<** 0.001), indicating that CDK5 up-regulation may be relative to the carcinogenesis and progression of cervical cancer. We also investigated the corrections between CDK5 and clinicopathological variables. However, these clinicopathological variables, such as pathological grade, lymph node metastasis and FIGO stage above only showed weak relevance with SCC, thus, the relationship between CDK5 expression and the clinicopathological parameters needs to be confirmed with larger sample size.

Currently, there are merely two researches on CDK5 in cervical cancer. A study reported that CDK5 had a role in decreasing the growth of human cervical cancer cell line when retinoic acid treatment was applied to use and the expression of CDK5 and p35 were up-regulated by retinoic acid treatment [12]. So the study conferred CDK5 and p35 could have a positive effect on the retinoic acid-induced HeLa apoptosis [12]. Furuya et al. [24] also performed a study on HeLa cells and showed that CDK5 was linked to endocytic sorting and autophagy. CDK5/p25 is able to phosphorylate vacuolar protein sorting 34 (Vps34) at Thr159 site, which hinders Vps34 interaction with Beclin-1 during mitosis and finally blocks the autophagy. But there is no available research about CDK5 expression level and mechanisms in cervical cancer tissues and precancerous lesions. The genesis of cancer was closely correlated with abnormal regulation of DNA damage and repair. A research stated that CDK5 was mandatory for the DNA damage response in tumor cells [25] and another study showed that CDK5/STAT-3 oncogenic pathway played a key role in the expression of DNA repair genes [26].

Besides the expression of CDK5 in cervical cancer tissues, we further investigated the correlation between CDK5 expression and clinicopathological factors, which could reflect the deterioration and development of cervical cancer. For instance, higher CDK5 expression was found to be correlated with advanced FIGO stage, advanced TNM stage and lymph node metastasis. These results indicated that CDK5 might be a potential predictor for the deterioration and development of cervical cancer. Similar trend was reported in non-small cell lung cancer that CDK5 expression was associated with differentiation, lymph node metastasis and overall survival [20]. Thus, CDK5 may play consistent oncogenic roles in cervical carcinoma and non-small cell lung cancer. However, the role of CDK5 expression in the survival estimation needs further investigation.

Further, the results showed that higher CDK5 expression in cervical cancer with lymph node metastasis has some similarities with studies in other malignancies. For example, Liu et al. [20] examined CDK5 expression in 95 patients with non-small cell lung cancer (NSCLC). They found no association between CDK5 expression and clinicopathological features, such as the time of surgery, age, and gender of the patients or histopathology grading. However, significant correlations were observed between CDK5 expression and the degree of differentiation, the pathological stage, and lymph node metastases. More importantly, patients with CDK5-positive had a poor 5-year overall survival compared to CDK5-negative patients in NSCLC. CDK5 was reported to be involved in the regulation of proliferation and survival of breast cancer cells, and CDK5 was characterized as a downstream target of extracellular regulated protein kinases in carboplatin-induced cell death [27, 28]. The usage of shRNA technology or inhibitors to block the expression of CDK5 in pancreatic cancer cells could dramatically inhibit the growth of MIApaCa-2 cells, thereby reducing the incidence of tumor development [29]. Further, CDK5 controlled cell growth, migration and metastasis, and was indirectly involved in the regulation of cell cycle, which might contribute to the development of prostate cancer [30]. Besides, the inhibition of CDK5 either via shRNA or by its pharmacological inhibitor roscovitine could reduce the migration and growth of medullary thyroid carcinoma cells in vitro [31]. We conjectured that the role of CDK5 involved in the proliferation, migration and apoptosis of other tumors cells may provide clues to the ascertainment of its contribution in cervical cancer.

Our results showed that CDK5 expression had a close relationship with lymph node metastasis (*r* = 0.317, *P* **<** 0.001). The mechanism of CDK5 influencing the lymphatic metastasis remains unknown in cervical carcinoma. However, some similar studies in other disease could provide a hint. Oncogenic epithelial–mesenchymal transition (EMT) refers to the process in which epithelial malignant cells acquire mesenchymal cell phenotype, including enhanced migratory capacity and invasiveness, and is generally accepted as a mechanism underlying metastasis in many types of cancers [32], and so as in cervical cancer [33]. High expression of CDK5 or p35 had been demonstrated to be involved in the cancer cell motility and metastasis potential via EMT, such as in breast cancer [21], lung cancer [34], and head and neck squamous cell carcinoma [35]. In cervical cancer, the expression of miR-21 was up-regulated [36]. MiR-21 plays an important role in regulating biological behaviors in many malignancies. And miR-21 is associated with tumor biological behaviors, including apoptosis [37], lymph node metastasis [38], EMT [39], migration and invasion [34]. Study has shown that the expression of miR-21 and CDK5 were correlated with lymph node metastasis in head and neck squamous cell carcinoma [35]. In breast cancer, miR-21/CDK5 axis was abnormal activated which had an association with lymph node metastasis [38]. Though expression of CDK5 was detected in cervical cancer tissue and regulative mechanism of CDK5 was investigated in other cancers, the probable mechanism between CDK5 and lymph node metastasis in cervical cancer still needs further confirmation.

Based on our observations, we speculated that the up-regulated expression of CDK5 from precancerous lesions to cervical cancer tissues might be intimately correlated with the development of cervical carcinoma.

## Conclusions

In summary, the present study confirmed a higher expression level of CDK5 in cervical cancer tissues compared with precancerous lesions. These results may break new ground in the exploration of biologic behavior in cervical cancer. And CDK5 may become a new biomarker or molecular target for the treatment of cervical cancer. The clinical role of CDK5 in cervical cancer may be better defined with a larger sample size, and a long follow-up prognosis analysis is extraordinarily necessary. Therefore, future in vitro and in vivo studies to explore the carcinogenesis mechanism of CDK5 in cervical cancer is warranted. CDK5 may play an essential role in the development of cervical cancer and it may be useful for the clinical diagnosis, treatment and prognosis evaluation of cervical cancer.

## Abbreviations

CDK5: cyclin-dependent kinase5
IHC: immunohistochemistry
CA: condyloma acuminate
SCC: squamous cell carcinoma
HPV: human papilloma virus
FIGO: The International Federation of Gynecology and Obstetrics
WHO: World Health Organization
TNM: tumor node metastasis
AJCC: American Joint Committee on Cancer
RA: retinoic acid
NSCLC: non-small cell lung cancer
